# AI-Guided Remission: Protocol for a Conversational Agent (Chatbot) for Dosing Activity and Footwear Progression After Diabetic Limb Reconstruction

**DOI:** 10.3390/s26082299

**Published:** 2026-04-08

**Authors:** Lucian M. Feraru, David C. Klonoff, Bijan Najafi, Magdalena Antoszewska, David G. Armstrong

**Affiliations:** 1 Department of Podiatric Surgery, Adventist Health White Memorial, Los Angeles, CA 90033, USA; 2 Diabetes Research Institute, Mills-Peninsula Medical Center (Sutter Health), San Mateo, CA 94401, USA; dklonoff@diabetestechnology.org; 3 Center for Advanced Surgical and Interventional Technology (CASIT), David Geffen School of Medicine, UCLA, Los Angeles, CA 90095, USA; bnajafi@mednet.ucla.edu; 4 Department of Dermatology, Venereology and Allergology, Medical University of Gdańsk, 7 Dębinki St., 80-211 Gdańsk, Poland; mmantoszewska@gmail.com; 5 Department of Surgery, Keck School of Medicine, University of Southern California, Los Angeles, CA 90033, USA; armstrong@usa.net

**Keywords:** diabetic foot, remission, activity dosing, thermometry, footwear, chatbot, artificial intelligence

## Abstract

**Highlights:**

**What are the main findings?**

- A conversational agent (chatbot) can translate diabetic foot remission guidelines into personalized daily activity dosing and footwear progression protocols, informed by clinical expertise and evidence-based practice.
- The system uses a closed-loop approach integrating step counts, foot thermometry, and footwear tracking to dynamically adjust patient activity targets, aligned with clinician-guided decision rules.

**What are the implications of the main findings?**

- AI-guided remission care can extend specialist oversight into patients’ homes during the critical post-healing period when recurrence risk is highest, leveraging expert-informed care pathways.
- This protocol evaluates whether automated daily monitoring with personalized guidance to improve treatment adherence is feasible and safe based on established clinical principles, with clinical efficacy to be tested in subsequent trials.

**Abstract:**

Background: Diabetic foot ulcers recur frequently after healing. The first three months carry the highest risk. Remission is a vulnerable phase that demands precise self-care and timely feedback. Evidence supports thermometry and protective footwear with gradual return to activity, yet adherence at home is inconsistent. Objective: To describe the design and planned evaluation of a conversational agent (chatbot) that guides patients through the remission phase following diabetic limb reconstruction. Methods: This protocol describes a conversational agent (chatbot) that turns remission guidance into daily actions, grounded in clinical expertise and established care guidelines. Walking is dosed like a drug, with careful titration based on tissue response. The agent integrates automatic data capture (smartphone step counts, skin temperature, shoe step streams, smartwatch step streams, Bluetooth thermometry when available, and app session timestamps) with manual patient entries (shoe wear time, skin redness persistence, and symptom checks). It doses walking activity, guides footwear break-in, prompts photo-confirmed concerns, following clinician-informed rules and escalation pathways. We define data quality checks for missingness and physiologic plausibility, and the agent reinforces reducing weight-bearing activity when risk signals appear. We outline device drift. The study is designed as a single-arm feasibility pilot (*n* = 30) to assess engagement, safety, and implementation fidelity. Results: No clinical outcome results are reported because this is a protocol study and enrollment has not yet begun. This study presents the prespecified sensing-to-decision workflow, escalation logic, and pilot endpoints, along with internal technical verification procedures (e.g., message delivery reliability, data completeness checks, and rule-engine consistency testing). Conclusions: A remission chatbot is a plausible method to extend specialist support into the home, reflecting integration of clinical expertise with digital health tools. This protocol defines how feasibility, safety, and usability will be evaluated. Clinical efficacy should be confirmed in future studies.

## 1. Introduction

Diabetes-related foot ulcers are a leading cause of disability, hospitalization, and lower extremity amputation worldwide. The global prevalence of diabetic foot ulcers is estimated at 6.3%, with lifetime incidence reaching 19–34% among individuals with diabetes [1]. Recurrence is alarmingly common even after successful wound healing, with studies reporting rates of 40% within one year and up to 65% within five years [1]. The first three months following ulcer healing represent a period of heightened vulnerability, as newly epithelialized tissue remains fragile and susceptible to mechanical stress [1,2].

The concept of diabetic foot remission, analogous to cancer remission, has emerged as a framework for understanding this critical post-healing phase [1]. Remission is not synonymous with cure; rather, it represents a state requiring ongoing vigilance, structured self-care, and timely clinical intervention. The primary goal during remission is to maximize ulcer-free days by preventing recurrence through safe tissue loading, protective footwear, and early detection of tissue stress [1,2]. Home-based temperature monitoring has emerged as an evidence-based strategy for detecting impending tissue breakdown before clinical ulceration occurs. Elevated skin temperature at specific foot locations, typically defined as a side-to-side difference of 2.2 °C (4 °F) or greater, signals subclinical inflammation and predicts ulcer development [3,4,5,6]. Multiple randomized controlled trials have demonstrated that daily temperature monitoring combined with activity modification when hotspots appear can reduce ulcer recurrence by 40–70% in high-risk patients [3,4,5,6]. This preventive approach represents a paradigm shift from reactive wound care to proactive tissue protection.

Guidelines from the International Working Group on the Diabetic Foot (IWGDF) provide comprehensive recommendations for ulcer prevention, including offloading, therapeutic footwear, regular foot checks, patient education, and integrated care [2]. However, translating these evidence-based recommendations into consistent daily actions remains challenging for patients and clinicians alike. Studies report that adherence to offloading devices ranges from 15 to 60%, with significant day-to-day variability influenced by comfort, convenience, and patient understanding of risk [7,8]. This gap between guidelines and implementation represents a critical opportunity for technology-enabled solutions.

Artificial intelligence-based conversational agents, commonly known as chatbots, offer a promising approach to bridging this implementation gap. These systems can be deployed on mobile platforms to deliver personalized advice, provide reminders, capture health data, and support secure messaging with clinical teams [9]. These systems are increasingly appealing to patients and clinicians because they can provide tailored medical support precisely at the point of need [10]. By providing interactive advice and answering user queries, they may offer advantages over static online resources, such as greater personalization and engagement [11]. The conversational format mimics human interaction, potentially increasing patient comfort and willingness to share health concerns.

AI chatbots have shown a mean accuracy exceeding 90% in providing answers to clinical questions related to diabetic foot ulcers based on clinical guidelines [12]. Patients with diabetic foot ulcers may need frequent treatment adjustments based on wound status, activity tolerance, and symptom evolution [13]. To our knowledge, no chatbot has been reported for patients with diabetic foot ulcers that can dose activity, track temperature, evaluate symptoms, and provide timely treatment adjustments. This study presents a protocol describing the design, system architecture, and planned feasibility evaluation of such a conversational agent. The contribution of this work lies in the development of a structured, closed-loop, sensor-driven framework with prespecified decision logic, escalation pathways, and evaluation metrics to support diabetic foot remission management, without reporting clinical outcomes at this stage.

## 2. Methods

### 2.1. System Overview

**Figure 1:**
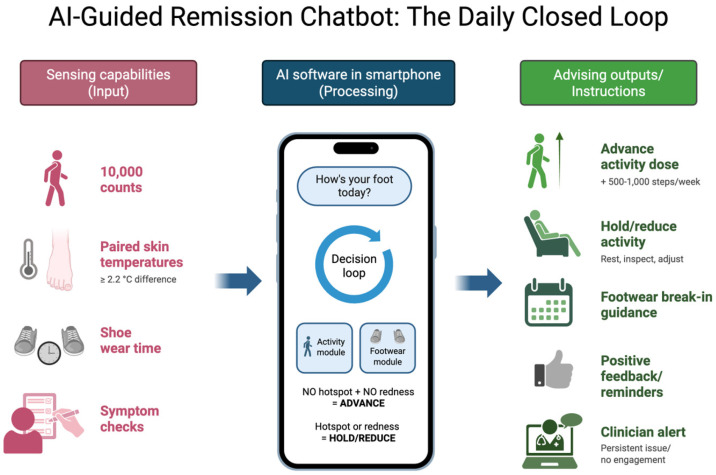
Daily dosing decision loop. Expanded sensing-to-decision workflow for the remission chatbot, showing automatic and manual inputs, data-quality checks, green/amber/red risk tiers, recommendation outputs, and clinician escalation pathways.

The chatbot runs on a mobile platform with secure messaging, reminders, and structured data capture. It prompts daily check-ins and can send on-demand alerts when sensor data or user reports suggest tissue stress. The system architecture consists of four integrated layers: [1] a patient-facing mobile application providing the conversational interface; [2] a secure cloud backend for data storage and processing; [3] sensing integration modules for wearable sensors and temperature monitoring devices; and [4] a clinical dashboard enabling care team oversight. The sensing layer accepts smartphone pedometer data, wearable step feeds (e.g., Apple Health/Fitbit class devices), and thermometry inputs from handheld or connected infrared devices. Data are timestamped, range-checked, deduplicated, and tagged as automatic versus manual entries before rule execution. Missing data trigger reminder prompts and conservative decision defaults. All data transmission uses end-to-end encryption (in transit and at rest) with healthcare role-based access controls. Figure 1 illustrates ingestion, data quality checks, risk stratification, recommendation output, and escalation handoffs.

The sensor subsystem acquires data at multiple resolutions. Step counts are captured via smartphone accelerometers or paired wearable devices at 25–100 Hz raw accelerometry, aggregated into epoch-level counts at one-minute resolution. Foot skin temperature is measured with infrared thermometry sensors (resolution 0.1 degrees C, accuracy ±0.3 degrees C) at standardized bilateral plantar sites, with the patient guided through a consistent measurement protocol to reduce operator variability. Shoe wear time is logged through in-shoe pressure sensors or accelerometers that detect donning and doffing events. Data preprocessing includes median filtering to remove motion artifacts from temperature readings, step-count validation against minimum bout duration (at least 10 consecutive steps) to exclude incidental movements, and gap-filling algorithms for brief sensor dropouts (less than 15 min) using linear interpolation. Missing data exceeding 15 min are flagged rather than imputed, and the chatbot prompts the patient to re-measure. Reliability is maintained through automated daily sensor health checks, battery-level monitoring, and Bluetooth connectivity verification, with alerts issued if any data stream is absent for more than four hours during waking periods.

### 2.2. Decision Logic

The agent applies two linked modules (Figure 1). First, the footwear module manages break-in by prompting patients through graduated wear schedules while monitoring and post-wear checks for signs of poor fit or pressure-related injury [14]. The footwear module classifies patients into three categories based on their break-in status: states: [1] actively breaking in new footwear, requiring daily wear-time tracking and post-wear inspection prompts; [2] full transition to stable therapeutic footwear, requiring periodic adherence checks; or [3] needing footwear modification or replacement, triggering clinical escalation. Second, the activity module adjusts daily step targets using thermometry, symptom checks, and adjusts targets based on temperature reading milestone status. Formal daily rule sequence: (a) ingest prior 24 h sensor and clinical milestones; (b) validate data quality and missingness; (c) assign risk tier (green/amber/red); (d) issue next-day step and footwear recommendation; (e) trigger escalation when red criteria persist or safety flags are present; (f) log rationale for clinician audit.

Thermometry uses a side-to-side difference threshold of about 2.2 °C or 4 °F (4 °F) at matched plantar sites. If the difference exceeds this threshold for one day, a caution and state with reduced activity is recommended by the chatbot. If it persists for two or more days, the system escalates to the consecutive days and alerts the clinical team with an escalation and recommendation for direct evaluation. This threshold is supported by prior randomized controlled trials demonstrating that sustained temperature asymmetry predicts ulceration, and is implemented with guardrails including repeated-measure confirmation prompts and checks for outlier values suggestive of sensor or user error. The chatbot guides patients through standardized measurement protocols to ensure consistent, reliable readings and flags potential measurement errors for clinician review when data quality is uncertain.

### 2.3. Footwear Break-In Protocol

Patients transition into custom therapeutic footwear with a structured wear-in plan. A typical plan begins with 30 to 60 min of wear on Day 1, increasing by one to two hours daily while inspecting the feet before and after each session. If redness persists beyond about 30 min or if any skin breakdown appears, progression pauses, the clinical team is alerted, and shoe fit is adjusted [15,16]. The break-in protocol recognizes that even well-fitted therapeutic footwear requires gradual accommodation to prevent pressure-related tissue damage during the vulnerable remission period. Patients are instructed to perform visual inspection of their feet after removing shoes, checking for redness, blisters, or areas of warmth. Table 1 shows the recommended break-in schedule.

### 2.4. Activity Dosing Protocol

Once full-day shoe use is tolerated, walking is dosed like a drug. Patients begin near baseline step counts, defined as the median daily step count over a 7-day run-in period with no red-flag thermometry events, and advance weekly by increments of roughly 500 steps per day. Weekly advancement is ~500 steps/day when temperature differences remain within the normal threshold and no persistent skin redness is observed. If a hotspot emerges, the chatbot recommends temporary step reduction and intensified monitoring until normalization. The app compares measured versus prescribed activity until temperatures normalize. This graduated approach allows controlled tissue loading while monitoring for signs of stress. The dosing paradigm treats physical activity as a therapeutic intervention requiring careful titration based on individual response. The chatbot tracks daily step counts through smartphone sensors or connected wearables and compares actual activity against prescribed targets, providing feedback. Table 2 shows the activity dosing progression.

### 2.5. Gait and Mobility Framing

Baseline mobility informs starting goals. Patients with limited community ambulation start lower and progress more slowly. Those who regularly exceed 5000 steps may begin at higher baselines. The system adjusts thresholds if wearable gait data indicate limping, asymmetry, or unusual patterns that could signal overloading or compensatory mechanics [17,18,19]. Gait quality metrics, when available from instrumented insoles or smartphone sensors, can enrich monitoring by detecting subtle changes in walking patterns that may precede clinical symptoms. The chatbot incorporates these data streams when available, using them to refine activity recommendations and identify patients who may benefit from gait training or assistive devices.

### 2.6. Pilot Study Design

Table 3 summarizes the key study design components. Design: Single-arm feasibility trial over 12 weeks. Population: 30 adults with diabetes in remission after ulcer healing or reconstructive surgery, with healed skin at enrollment. Inclusion criteria: ≥18 years, type 1 or type 2 diabetes, history of diabetic foot ulcer healed within the past 6 months, and access to a smartphone. Exclusion criteria: Active infection, critical limb ischemia, cognitive impairment limiting capacity for informed consent, or inability to perform self-monitoring. Intervention: Chatbot-guided daily self-monitoring, with the chatbot providing daily guidance and recommendations. Visits: Clinic checks at weeks 4, 8, and 12 with comprehensive foot examination. Sample-size rationale: *n* = 30 is appropriate for feasibility assessment and allows estimation of completion, retention, and safety event rates to inform go/no-go decisions for a future randomized trial.

The primary outcomes are feasibility and safety. Feasibility endpoints include the proportion of days with completed check-ins (target ≥ 80%) and 12-week retention (target ≥ 80%). Safety outcome endpoints include ulcer recurrence during the study period, device-related adverse events, and unplanned hospital utilization. Escalation framework: Amber status prompts same-day self-management reinforcement and repeat check; red status (persistent temperature asymmetry, concerning symptom cluster, or reported skin breakdown report) prompts clinician notification, chart flag, and any unplanned hospitalizations. The study will track whether patients who receive chatbot-guided care demonstrate different patterns of clinical contact compared to historical controls, including frequency of urgent visits and time to first post-healing complication. All serious adverse events will be are reviewed by an independent safety monitor.

Secondary outcomes include change in steps per day from baseline, number of hotspot alerts, unscheduled visits, and patient-reported usability. The System Usability Scale (SUS) will be administered at study completion to assess user experience. Qualitative interviews will explore patient perceptions of the chatbot’s usefulness, barriers to engagement, and suggestions for improvement. Caregiver involvement and family engagement will also be assessed, recognizing that diabetic foot care often involves support networks beyond the individual patient. Economic outcomes including healthcare utilization and costs will be captured for future cost-effectiveness analyses.

## 3. Results

No clinical efficacy results are reported in this manuscript because this is a protocol study and enrollment has not yet started. The protocol study is currently in preparation, with planned enrollment beginning in early 2026. This study defined the prespecified analyses and technical framework that will be evaluated at study completion, including engagement rates, alerts, alert burden, protocol adherence, safety signals, and early safety signals from the feasibility trial. In addition to outlining planned clinical and usability endpoints, this study reports the system architecture, sensing-to-decision workflow, escalation logic, and predefined evaluation metrics, which constitute the primary contribution of this protocol. Preliminary usability testing with a small group of patients has been conducted to refine the chatbot interface (including SUS). We also prespecified internal technical validation procedures prior to enrollment, including message delivery success rate, sensor-data completeness, rule-engine reproducibility across replayed test cases, and conversation flows. Initial feedback suggests high acceptability of the daily check-in format and positive reception of the personalized activity recommendations. Full results including quantitative engagement metrics, temperature alert patterns, patient-reported outcomes and clinician adjudication concordance for escalation triggers will be reported following completion of the 12-week pilot study.

## 4. Discussion

AI-based chatbots and digital reminders have the potential to promote healthy behaviors by delivering personalized guidance at scale. In the context of diabetic foot remission, where consistent daily actions are critical to prevent recurrence, a conversational agent can bridge the gap between clinic visits and home-based self-management. The chatbot approach leverages the ubiquity of smartphones and the growing acceptance of digital health tools among patients with chronic conditions. Unlike static educational materials, conversational agents can adapt their messaging based on patient responses and sensor data, creating a dynamic support system that evolves with the patient’s needs.

An AI remission assistant can translate guidance recommendations into simple daily actions, transforming complex clinical protocols into manageable patient tasks. Thermometry provides an early warning signal that may support earlier offloading with clinician oversight before tissue damage becomes clinically apparent. The closed-loop nature of the system—collecting data, analyzing patterns, providing feedback, and escalating when needed—creates a continuous workflow (capture → classify → recommend → escalate) that is explicitly defined in this protocol to standardize home monitoring and extend specialist oversight between visits. This approach may be particularly valuable and aligns with recent proposals to embed AI as an early warning layer within DFU telemonitoring programs, where operational triage can protect clinician attention for patients living with deteriorating wounds while stable trajectories remain in rural areas or those with limited access to specialized diabetic foot clinics, potentially reducing geographic disparities in low-burden remote care quality [20].

Sustained engagement is critical. The agent uses concise prompts, positive feedback, and adaptive frequency to maintain user interaction while minimizing alert fatigue. Evidence suggests earlier detection can improve outcomes [21,22], though alert fatigue remains a risk for concern [23]. Key practical advantages include (1) continuous structured monitoring, (2) scalable reinforcement when implementing chatbot self-care behaviors, and (3) improved triage visibility for diabetic foot remission care, including (1) continuous, data-driven support; (2) scalability teams. However, important risks must be acknowledged, including missed emergencies, over-trust in automation, alert fatigue, privacy exposure, and engagement; and (3) access and workflow benefits. A data-driven chatbot reacts to the inequitable usability of the environment in near real time, which aligns with randomized controlled trial evidence that home temperature monitoring and telemedical systems can predict and prevent diabetic foot ulcer recurrence when acted upon promptly [3,4,5,6]. The chatbot can integrate frequent foot temperature readings and activity/adherence data and respond dynamically. Automated pattern recognition can trigger earlier offloading advice or clinical escalation than usual clinic schedules, potentially reducing ulcer recurrence. Diabetes chatbots have improved glycemic outcomes and self-management behaviors in multiple trials, with good user acceptance and reduced out-of-hospital workload [9,10,11]. They can deliver tailored education—explaining why a temperature alert matters, how to offload, and footwear tips—in short and repeated interactions that reinforce learning. Chatbots can provide 24/7 first-line guidance, reinforce clinician instructions, and collect structured symptom data that can be reviewed by the diabetic foot care team, supporting telehealth models already shown to be feasible for diabetic foot monitoring [24,25]. Despite these advantages, several categories of risk must be acknowledged. General studies of conversational assistants show that a substantial fraction of medical recommendations could lead to harm, including failure to recognize emergent symptoms [26,27]. Patients may over-trust the chatbot, particularly in low-digital-literacy populations [26,27,28,29,30,31,32,33], and accordingly delay seeking in-person care. Overly sensitive thresholds could generate excessive alerts, causing alarm fatigue and reduced adherence [28,29]. Privacy and cybersecurity risks could expose sensitive health information [30,31]. Older adults or those with low health literacy may find sensor-plus-chatbot systems difficult to use, potentially widening disparities [32,33]. Accordingly, in this protocol the chatbot is explicitly designed as decision support rather than autonomous diagnosis, with predefined escalation pathways that require clinician oversight for high-risk signals. It is intended as an adjunct to care, not a replacement, and encourages in-person or telehealth evaluation when needed.

As a protocol study, this work focuses on feasibility rather than clinical effectiveness. If acceptable and safe, the next step is a randomized trial that compares standard care to standard care plus the chatbot. Key endpoints would include chatbot-guided remission support, with ulcer recurrence and time-to-event outcomes at 6 to 12 months and time to event with health economic measures and quality of life outcomes as secondary endpoints. The chatbot design allows for iterative refinement based on patient feedback, enabling continuous improvement of the user experience and clinical algorithms. Future development may incorporate machine learning to personalize thresholds and recommendations based on individual patient patterns. Regulatory and ethical issue priorities include privacy, security, clinical clinician oversight, and transparency of recommendation logic. The system is intended to provide decision support that augments clinician judgment rather than replacing professional medical care. The algorithm and thresholds are documented for peer review and can be adapted as evidence evolves.

The chatbot’s recommendation logic is designed to be interpretable, allowing clinicians to understand and verify the basis for any advice given to patients.

The trajectory from conversational agents to physical robotic systems is already becoming visible. In January 2026, the United States Centers for Medicare and Medicaid Services highlighted Alabama’s plan to deploy telerobotic ultrasound systems in maternity care deserts where no obstetricians practice, allowing sonographers to perform examinations from remote locations. Early evidence from rural northern Norway demonstrates that robot-assisted obstetric ultrasound achieves excellent diagnostic reliability and high patient satisfaction compared with traditional examination [34]. These developments suggest that the chatbot described here may eventually serve as the conversational front end for a broader ecosystem of remotely operated diagnostic and therapeutic devices, extending not only monitoring but also physical examination into the home. This trajectory—from conversational AI to remotely operated physical systems—reflects a broader convergence of regenerative medicine, human–machine interfaces, and personalized health monitoring with human-centered AI, as recently described [35].

## 5. Limitations

The pilot is small and short. Some protocol elements are based on consensus and guidelines rather than high-grade evidence, limiting the ability to draw definitive conclusions about optimal thresholds or intervention timing. The temperature threshold of 2.2 °C is supported by randomized trials but may require individualization based on patient characteristics such as baseline temperature variability, ambient conditions, and measurement technique [36]. The chatbot cannot yet interpret images, and certain high-risk findings require clinical judgment that exceeds current AI capabilities [37]. The study population may not be representative of all patients with diabetic foot disease, particularly those with limited digital literacy or access to smartphones. Integration with telemedicine plus at-home temperature monitoring is feasible but access remains limited [20]. Future iterations may incorporate image analysis, more sophisticated gait assessment, and integration with continuous glucose monitoring systems.

## 6. Conclusions

A remission chatbot may help operationalize evidence-based self-care during the high-risk post-healing phase. In this protocol, multimodal sensor streams are combined with structured patient interaction to extend specialist oversight into the home. The system provides consistent monitoring, personalized recommendations, and timely escalation when concerning signs emerge. As this is a protocol study, definitive claims regarding clinical effectiveness are premature and will be evaluated in the planned pilot and subsequent trials. This approach has the potential to improve patient engagement and support scalable remission care.

## Figures and Tables

**Table 1:** Footwear break-in protocol schedule.

Day	Wear Duration	Post-Wear Inspection	Advancement Criteria
1	30–60 min	Check for redness	No redness → advance
2	1–2 h	Check for redness	Redness resolves <30 min
3	2–3 h	Check for redness	Redness resolves <30 min
4	3–4 h	Check for redness	Redness resolves <30 min
5	4–6 h	Check for redness	Redness resolves <30 min
6	6–8 h	Check for redness	Redness resolves <30 min
7+	Full day	Daily inspection	Monitor ongoing tolerance

**Table 2:** Activity dosing progression schedule.

Week	Target Steps/Day	Advancement Criteria
1	Baseline (1000–1500)	Establish baseline
2	Baseline + 500	No hotspots
3	Baseline + 1000	No hotspots
4	Baseline + 1500	Clinic visit
5–8	Baseline + 2000–2500	Week 8 visit
9–12	Individualized	Maintenance goals

**Table 3:** Study design components.

Component	Description
Design	Single-arm prospective pilot
Population	Adults with diabetic post-limb reconstruction (Charcot, osteomyelitis, or chronic wound); remission phase
Intervention	Daily chatbot interaction via SMS for 90 days post-discharge
Outcomes	Ulcer recurrence, adherence to activity/footwear protocol, patient satisfaction
Sample Size	Target *n* = 30 for feasibility assessment

## Data Availability

No datasets were generated or analyzed during this study. This is a protocol paper describing a planned intervention.

## References

[1] Armstrong D.G., Boulton A.J.M., Bus S.A. (2017). Diabetic Foot Ulcers and Their Recurrence. N. Engl. J. Med..

[2] Bus S.A., Lavery L.A., Monteiro-Soares M., Rasmussen A., Raspovic A., Sacco I.C.N., van Netten J.J. (2020). Guidelines on the prevention of foot ulcers in persons with diabetes (IWGDF 2019 update). Diabetes Metab. Res. Rev..

[3] Lavery L.A., Higgins K.R., Lanctot D.R., Constantinides G.P., Zamorano R.G., Armstrong D.G., Athanasiou K.A., Agrawal C.M. (2004). Home monitoring of foot skin temperatures to prevent ulceration. Diabetes Care.

[4] Bus S.A., Aan de Stegge W.B., van Baal J.G., Busch-Westbroek T.E., Nollet F., van Netten J.J. (2021). Effectiveness of at-home skin temperature monitoring in reducing the incidence of foot ulcer recurrence in people with diabetes: A multicenter randomized controlled trial (DIATEMP). BMJ Open Diabetes Res. Care.

[5] Armstrong D.G., Holtz-Neiderer K., Wendel C., Mohler M.J., Kimbriel H.R., Lavery L.A. (2007). Skin temperature monitoring reduces the risk for diabetic foot ulceration in high-risk patients. Am. J. Med..

[6] Rothenberg G.M., Page J., Stuck R., Spencer C., Kaplan L., Gordon I. (2020). Remote Temperature Monitoring of the Diabetic Foot: From Research to Practice. Fed. Pract..

[7] Crews R.T., Shen B.J., Campbell L., Lamont P.J., Boulton A.J., Peyrot M., Kirsner R.S., Vileikyte L. (2016). Role and Determinants of Adherence to Off-loading in Diabetic Foot Ulcer Healing: A Prospective Investigation. Diabetes Care.

[8] Littman A.J., Timmons A.K., Korpak A., Chan K.C., Jones K.T., Shirley S., Nordrum K., Robbins J., Masadeh S., Moy E. (2024). Remote Foot Temperature Monitoring Among Veterans: Large Observational Study of Noncompliance and Its Correlates. JMIR Diabetes.

[9] Aggarwal A., Tam C.C., Wu D., Li X., Qiao S. (2023). Artificial Intelligence-Based Chatbots for Promoting Health Behavioral Changes: Systematic Review. J. Med. Internet Res..

[10] Laymouna M., Ma Y., Lessard D., Schuster T., Engler K., Lebouche B. (2024). Roles, Users, Benefits, and Limitations of Chatbots in Health Care: Rapid Review. J. Med. Internet Res..

[11] Cevasco K.E., Morrison Brown R.E., Woldeselassie R., Kaplan S. (2024). Patient Engagement with Conversational Agents in Health Applications 2016-2022: A Systematic Review and Meta-Analysis. J. Med. Syst..

[12] Shiraishi M., Lee H., Kanayama K., Moriwaki Y., Okazaki M. (2024). Appropriateness of Artificial Intelligence Chatbots in Diabetic Foot Ulcer Management. Int. J. Low. Extrem. Wounds.

[13] Everett E., Mathioudakis N. (2018). Update on management of diabetic foot ulcers. Ann. N. Y. Acad. Sci..

[14] International Working Group on the Diabetic Foot (2019). IWGDF Practical Guidance on Prevention and Management of Diabetic Foot Disease.

[15] SafeStep Break-In Instructions for New Shoes. https://www.safestep.net/.

[16] Services AP Ankle & Foot Centers of America. Diabetic Shoes and Inserts Wear and Care. https://ankleandfootcenters.com/.

[17] Grewal G.S., Bharara M., Menzies R., Talal T.K., Armstrong D., Najafi B. (2013). Diabetic peripheral neuropathy and gait: Does footwear modify this association?. J. Diabetes Sci. Technol..

[18] Najafi B., Khan T., Fleischer A., Wrobel J. (2013). The impact of footwear and walking distance on gait stability in diabetic patients with peripheral neuropathy. J. Am. Podiatr. Med. Assoc..

[19] Najafi B., Mishra R. (2021). Harnessing Digital Health Technologies to Remotely Manage Diabetic Foot Syndrome: A Narrative Review. Medicina.

[20] Dardari D. (2026). Embedding Artificial Intelligence in Remote Follow-Up of Diabetic Foot Ulcers. J. Diabetes Sci. Technol..

[21] Lavery L.A., Higgins K.R., Lanctot D.R., Constantinides G.P., Zamorano R.G., Athanasiou K.A., Armstrong D.G., Agrawal C.M. (2007). Preventing diabetic foot ulcer recurrence: Temperature monitoring. Diabetes Care.

[22] Aan de Stegge W.B., van Netten J.J., Dijkgraaf M.G.W., Bus S.A. (2023). Cost-effectiveness of at-home temperature monitoring (DIATEMP). J. Diabetes Complicat..

[23] Ancker J.S., Edwards A., Nosal S., Hauser D., Mauer E., Kaushal R., HITEC Investigators (2017). Effects of workload and repeated alerts on alert fatigue. BMC Med. Inf. Decis. Mak..

[24] Hazenberg C.E., aan de Stegge W.B., Van Baal S.G., Moll F.L., Bus S.A. (2020). Telehealth for the diabetic foot: A systematic review. Diabetes Metab. Res. Rev..

[25] Smith-Strøm H., Igland J., Østbye T., Tell G.S., Hausken M.F., Graue M., Skeie S., Cooper J.G., Iversen M.M. (2018). Telemedicine follow-up for diabetes-related foot ulcers. Diabetes Care.

[26] Yau J.Y.-S., Saadat S., Hsu E., Murphy L.S.-L., Roh J.S., Suchard J., Tapia A., Wiechmann W., Langdorf M.I. (2024). Accuracy of Prospective Assessments of 4 Large Language Model Chatbot Responses to Patient Questions About Emergency Care: Experimental Comparative Study. J. Med. Internet Res..

[27] Shiferaw M.W., Zheng T., Winter A., Mike L.A., Chan L.-N. (2024). Assessing the accuracy and quality of artificial intelligence (AI) chatbot-generated responses in making patient-specific drug-therapy and healthcare-related decisions. BMC Med. Inform. Decis. Mak..

[28] Kassakian S.Z., Yackel T.R., Gorman P.N., Dorr D.A. (2017). Clinical decision support malfunctions in EHR. Appl. Clin. Inform..

[29] Klonoff D.C. (2015). Cybersecurity for Connected Diabetes Devices. J. Diabetes Sci. Technol..

[30] Williams P.A., Woodward A.J. (2015). Cybersecurity vulnerabilities in medical devices. Med. Devices.

[31] Owusu B., Gbaba S., Juste J., Bivins B., Baptiste D. (2024). Digital Health Literacy and Black Older Adults. Nurs. Open.

[32] Nagori A., Keshvani N., Patel L., Dhruve R., Sumarsono A. (2024). Electronic health Literacy gaps among adults with diabetes in the United States: Role of socioeconomic and demographic factors. Prev. Med. Rep..

[33] Saenz A.D., Centi A., Ting D., You J.G., Landman A., Mishuris R.G., Mass General Brigham AIGC (2024). Establishing responsible use of AI guidelines: A comprehensive case study for healthcare institutions. NPJ Digit. Med..

[34] Olsen I.P., Mannsverk C.A., Pulk A.B., Fagertun H., Fors M. (2025). Introduction of robot-assisted obstetric ultrasound in rural Northern Norway. Acta Obstet. Gynecol. Scand..

[35] Armstrong D.G., Najafi B., Gao W., Klonoff D.C., Liu C. (2025). Repair, regeneration, and replacement, revisited (redux). J. Diabetes Sci. Technol..

[36] Deda L.C., Goldberg R.H., Jamerson T.A., Lee I., Tejasvi T. (2022). Dermoscopy practice guidelines for use in telemedicine. NPJ Digit. Med..

[37] Littman A.J., Timmons A.K., Korpak A., Chan K.G., Jones K.T., Shirley S., Nordrum K., Robbins J., Masadeh S., Moy E. (2023). Evaluation of the Effectiveness of Remote Foot Temperature Monitoring for Prevention of Amputation in a Large Integrated Health Care System. Diabetes Care.

